# Ductal Carcinoma In Situ of the Breast

**DOI:** 10.1155/2012/123549

**Published:** 2012-07-18

**Authors:** Richard J. Lee, Laura A. Vallow, Sarah A. McLaughlin, Katherine S. Tzou, Stephanie L. Hines, Jennifer L. Peterson

**Affiliations:** ^1^Department of Radiation Oncology, Mayo Clinic, Jacksonville, FL 32224, USA; ^2^Department of General Surgery, Mayo Clinic, Jacksonville, FL 32224, USA; ^3^Department of Internal Medicine, Mayo Clinic, Jacksonville, FL 32224, USA

## Abstract

Ductal carcinoma in situ (DCIS) of the breast represents a complex, heterogeneous pathologic condition in which malignant epithelial cells are confined within the ducts of the breast without evidence of invasion. The increased use of screening mammography has led to a significant shift in the diagnosis of DCIS, accounting for approximately 27% of all newly diagnosed cases of breast cancer in 2011, with an overall increase in incidence. As the incidence of DCIS increases, the treatment options continue to evolve. Consistent pathologic evaluation is crucial in optimizing treatment recommendations. Surgical treatment options include breast-conserving surgery (BCS) and mastectomy. Postoperative radiation therapy in combination with breast-conserving surgery is considered the standard of care with demonstrated decrease in local recurrence with the addition of radiation therapy. The role of endocrine therapy is currently being evaluated. The optimization of diagnostic imaging, treatment with regard to pathological risk assessment, and the role of partial breast irradiation continue to evolve.

## 1. Introduction

Ductal carcinoma in situ (DCIS) of the breast is a complex pathologic entity in which malignant cells arise and proliferate within the breast ducts without invasion of the basement membrane. The increased use of screening mammography has led to a significant increase in the diagnosis of earlier stage breast cancers, including ductal carcinoma in situ. According to the Surveillance Epidemiology and End Results program (SEER) from 1975–2008, in situ breast cancers represented approximately 15% of all new breast cancer diagnoses in the United States [1]. DCIS consists of approximately 84% of all in situ disease, with lobular carcinoma in situ (LCIS) forming the bulk of the remainder. DCIS will account for approximately 27% of all newly diagnosed breast cancers or 77,795 new cases estimated in 2011 [2]. The age-adjusted DCIS incidence had increased an average of 3.9% annually from 1973 to 1983 and approximately 15% annually from 1983 to 2008 [3]. Since 2003, the incidence of DCIS has declined in women aged 50 years and older, while the incidence continues to increase in women younger than age 50 [4]. Overall, the rate of increase in incidence has been higher for DCIS than for any other type of breast cancer. As the incidence of DCIS increases, the treatment options continue to evolve.

In the past, DCIS was an uncommon disease that was routinely treated with mastectomy. However, with the increasing acceptance of breast conservation therapy for invasive breast cancers, initial attempts at breast-conserving surgery have also indicated a potentially acceptable treatment modality for DCIS [5]. Currently, several studies have shown breast conservation therapy to be effective for the management of DCIS. In 1983, 71% of cases were treated by mastectomy in contrast to only 33% in 2007 [6]. Today, mastectomy, lumpectomy followed by radiation therapy, and lumpectomy alone have all been advocated as management strategies for DCIS. Treatment selection for the individual patient with DCIS requires a clinical, mammographic, and pathological evaluation. A large proportion of women diagnosed with DCIS today are candidates for breast conservation, with relatively few absolute or relative contraindications due to toxicity concerns. With improvements in modern breast reconstructive techniques, mastectomy may be a more appealing alternative for individuals with anticipated poor cosmetic outcome as a result of breast-conserving surgery and radiation therapy. One factor affecting cosmesis may include a large surgical defect required to attain negative margins. Prior to the determination of a patient's suitability for breast-conserving therapy, a thorough evaluation to determine the extent and characteristics of the patient's disease is necessary. Patient preference will also play a role in the final treatment decision. We present this paper as an update to our previous review in 2009 [7].

## 2. Patient Evaluation

An adequate history and physical examination with evaluation of the patient's overall health should be performed. History assessment should include a personal or family history of malignancy, a breast cancer risk assessment including previous breast biopsies, history of abnormal mammograms, and the use of hormone replacement therapy or oral contraceptives. Other factors include nulliparity or late age at first birth, late menopause, and obesity in postmenopausal women [4]. Physical examination should document tumor size and location if palpable, nipple appearance, and the presence of nipple discharge. A thorough examination of the opposite breast and bilateral axilla should clinically confirm limited disease. The overall breast size and configuration should be taken into consideration for assessment of treatment options. 

In the past, most DCIS had presented as a palpable mass. Now, less than 10% of disease is palpable, with an abnormality found radiographically as the most common presentation and is found in approximately 20% of all screening mammograms [8]. DCIS may also present as pathologic nipple discharge with or without a mass or may be identified incidentally in a breast biopsy performed to treat or diagnose another abnormality. Patients who present with a palpable mass have a significantly higher potential for occult invasion, multicentricity, and local recurrence, than those who present with nonpalpable lesions [9, 10]. If left untreated, invasive breast cancer may develop in 30–50% of DCIS [11, 12]. The anatomic location of DCIS within the breast is not significantly different than invasive carcinoma. Most tumors were found in the upper outer quadrant (43.9%), then in the upper inner quadrant (9.0%), in the central quadrant (8.5%), in the lower outer quadrant (8.1%), and finally in the lower inner quadrant (6.9%) [13]. DCIS is rarely multicentric with radiologic and pathologic correlative studies of mastectomy specimens in patients with DCIS indicating only one multicentric lesion out of 82 mastectomy specimens [14]. 

## 3. Radiographic Evaluation 

**Figure 1:**
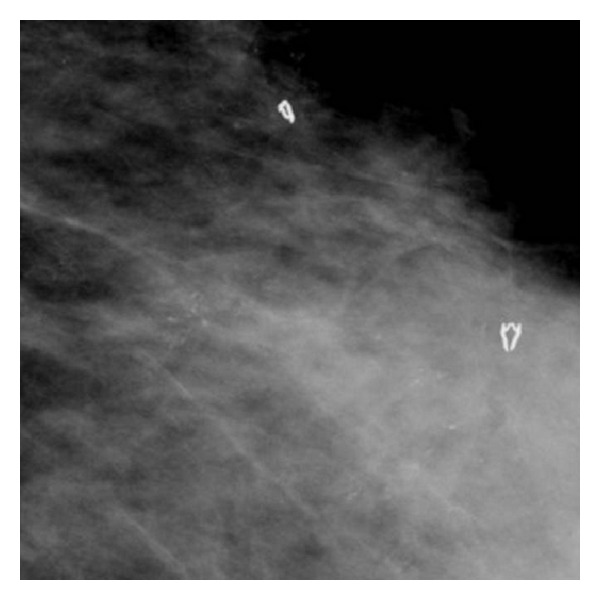
Mediolateral oblique projection of dystrophic branched calcifications.

**Figure 2:**
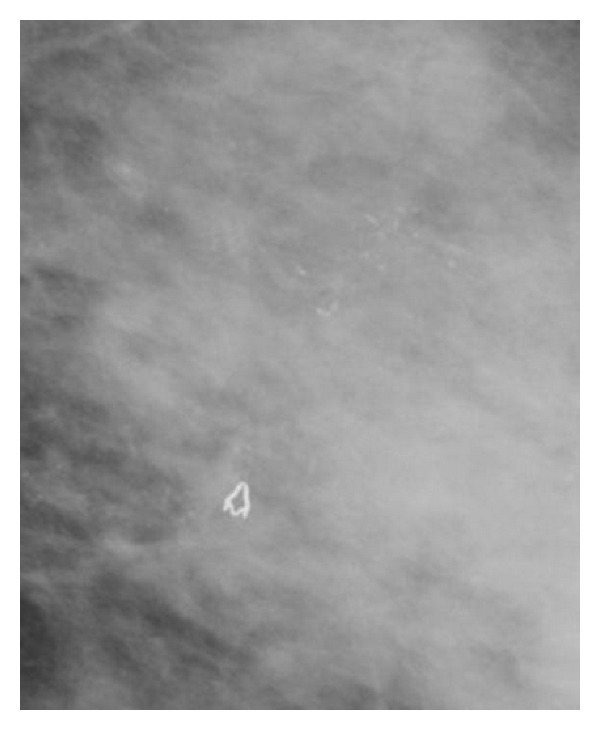
Craniocaudal view of calcifications with irregular shape and form.

**Figure 3:**
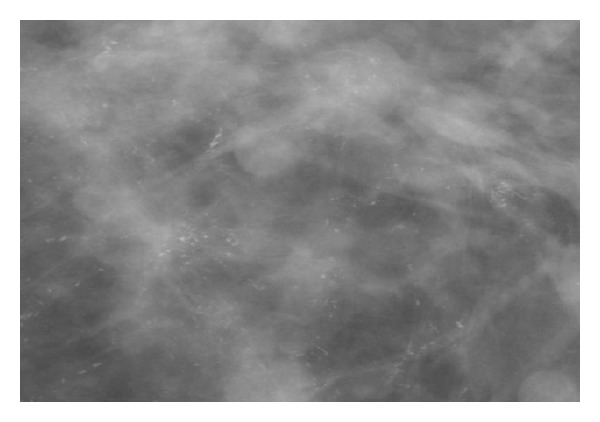
Extensive irregular pleomorphic calcifications with an underlying density.

DCIS commonly appears as clustered microcalcifications, although a nonpalpable mass may also represent DCIS. Calcifications are typically pleomorphic, varying in size, form, and density, and are grouped in segmental or linear arrangements reflecting their presence in the duct [15] (Figure 1). In contrast, calcifications associated with benign disease tend to be more rounded and uniform in density. Magnification views help delineate the presence and extent of microcalcifications (Figures 2 and 3).

The entire breast should be carefully examined to determine if areas of suspicion are present elsewhere in the breast. Mammography alone may underestimate the extent of disease. This is increasingly likely with larger lesions. Screening mammogram has an overall sensitivity of 55–86% [8, 16]. In a review of mammographically detected DCIS, 72% presented as calcifications and 12% presented as calcifications with an associated soft tissue abnormality [17]. Of malignant appearing microcalcifications, 92% are associated with a malignant histologic diagnosis [18]. All patients should have a mammogram performed before resection, and selected patients should have a mammogram performed after resection, in order to ensure the completeness of resection. Specimen radiography is helpful and may be performed routinely.

Breast MRI is currently being evaluated in DCIS. MRI has shown to be highly sensitive in the detection of invasive disease, with sensitivities ranging from 89–99%, but the sensitivity of MRI detection for DCIS is much lower, ranging from 40–80% [19]. Additionally, MRI can both under- and overestimate involvement, from 11–25% and 11–28%, respectively [20, 21]. In a multicenter study, the combination of mammography and MRI imaging has been shown to detect 82% of invasive lesions, but evaluation of the same dataset for DCIS showed that the combination of modalities was only able to detect 46% of DCIS due to a high false negative rate [19, 21]. This was still higher than the mammography alone detection rate of 35%. The increased sensitivity in the detection of occult multifocality and/or extensive residual disease [22–24] may help to guide local management decisions. Increased detection of breast abnormalities after MRI may alter the treatment management decision, with a change in treatment management in up to 15–28% of cases [21, 25]. 

**Figure 4:**
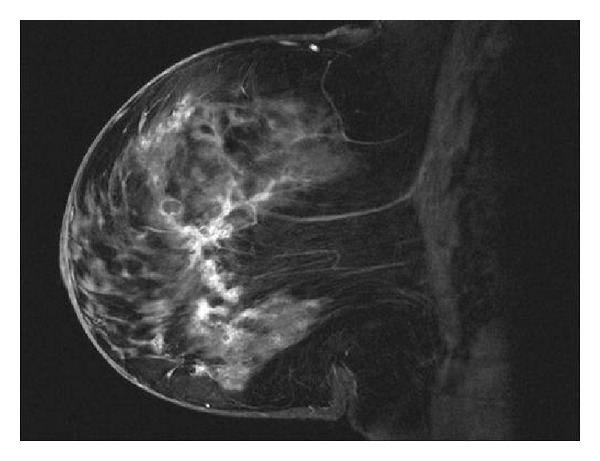
Abnormal enhancement of the ductal system.

The pattern of enhancement of DCIS in MRI can be variable including both ductal and regional enhancement (Figure 4). Ductal enhancement accounted for 21% of MRI detected lesions and 59% of 150 nonmass lesions [26]. Further study is currently underway to determine the optimal breast MRI technique for the identification of DCIS and to refine the histopathologic correlation [22, 24]. 

## 4. Diagnosis

As the majority of DCIS does not present with a palpable mass, image-directed procedures are necessary for diagnosis and treatment. Ultrasound-guided biopsy is useful for nonpalpable masses but usually cannot be relied upon for biopsy of microcalcifications. Stereotactic core needle biopsy may be used as the initial approach for biopsy of suspicious nonpalpable mammographic abnormalities with a sensitivity of 85–97% and a specificity approaching 100%. For the evaluation of microcalcifications, vacuum-assisted biopsy (VAB) is an even more accurate technique than core biopsy [27–29]. When possible, multiple cores should be taken and specimen radiography performed to confirm an adequate sampling of the abnormality. VAB is a reliable method to diagnose nonpalpable DCIS with a low underestimation rate and a false negative rate similar to that of open surgical biopsy [30]. Sensitivity and negative predictive value have been reported to be greater than 99% with VAB [27]. Open surgical biopsy is preferred if the abnormalities are not amenable to stereotactic breast biopsy or ultrasound-guided biopsy. For small lesions likely to be completely removed with the diagnostic biopsy, a marker should be left at the biopsy site for localization of the area. If a diagnosis of DCIS is made by percutaneous core needle biopsy, areas of invasion may be found in up to 20% of cases at the time of surgical excision [31].

## 5. Pathology

**Figure 5:**
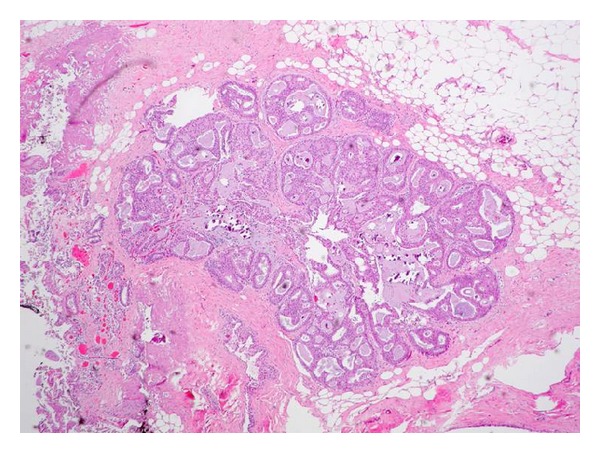
Low grade cribriform DCIS with associated microcalcifications.

**Figure 6:**
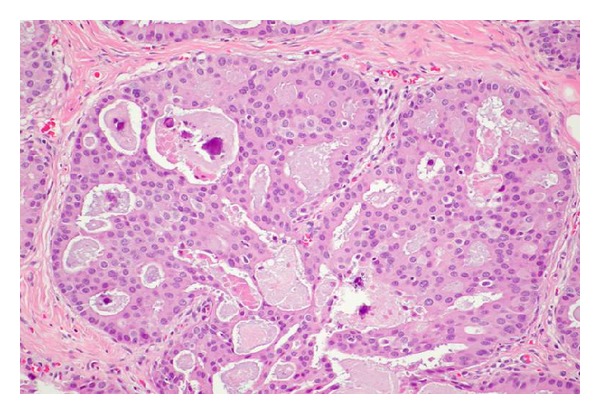
Cribriform DCIS with calcifications under high power magnification. Uniform even cell placement, central necrosis, and associated calcifications are seen.

**Figure 7:**
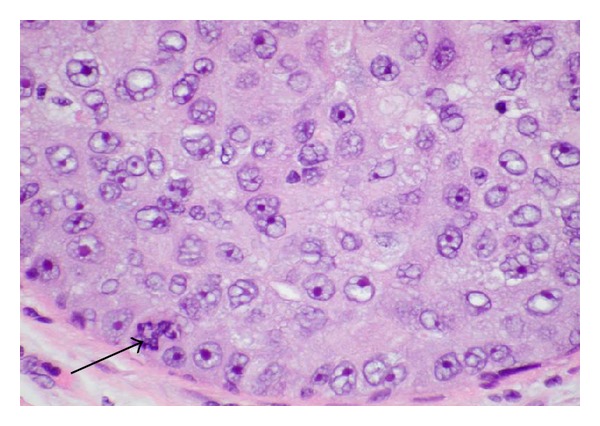
High-grade DCIS with atypical pleomorphic nuclei and prominent nucleoli seen under high power magnification. Bizarre (tripolar) mitotic figure at bottom left (arrow).

**Figure 8:**
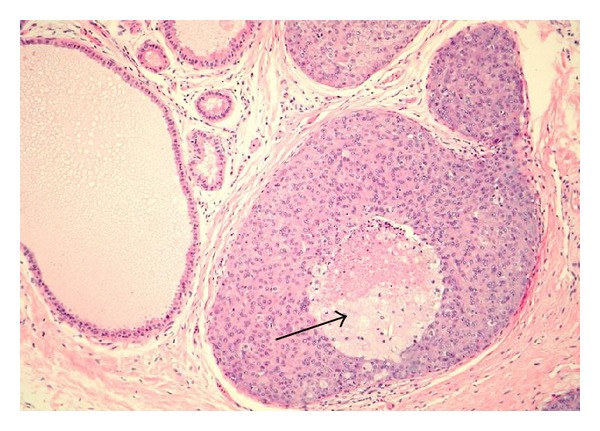
Solid DCIS with central necrosis (arrow). Adjacent benign ducts are shown for comparison.

DCIS is a heterogeneous entity with several morphologic variants that is thought to be part of a spectrum of proliferative ductal lesions of the breast that extend from epithelial hyperplasia without atypia to microinvasive carcinoma. DCIS had historically been classified primarily by architectural pattern into comedo, cribriform, papillary, micropapillary, and solid subtypes (several examples are seen in Figures 5, 6, 7, and 8). With the increasing use of breast conservation therapy, there is a need to identify those lesions more likely to recur or progress to invasive cancer, which are thought to correlate with tumors with higher nuclear grade and the presence of comedo necrosis [32]. More recently, there is a push to convert the designation of ductal carcinoma in situ to ductal intraepithelial neoplasia (DIN), similar to cervix cancer, although this has not been widely accepted [33].

The assessment of surgical margins is the most important detail in the pathologic evaluation of DCIS in patients under consideration for breast conservation. The definitions of positive and negative margins vary; however, microscopic extension to the surgical margin warrants further surgery [34]. Studies have shown that margins less than 1 mm show significant risk of recurrence [35], while there may not be additional benefit with a margin greater than 2 mm [36]. The pathologist should clearly specify whether DCIS is transected at the surgical margin and report the distance of the closest margin. 

## 6. Management of the Axilla

The incidence of lymph node metastases in DCIS is low, occurring in less than 1-2% of patients with DCIS [37] and is likely due to the presence of unrecognized invasive cancer [38]. The risk of finding occult invasive disease depends on how the cancer presented and on how the lesion was sampled. Although it is uncommon, DCIS may present as a palpable mass in up to 10% of all cases. These palpable DCIS may harbor invasive disease in up to 26% of cases. Further, when mastectomy is needed to treat the DCIS due to extensive calcifications throughout the breast, the risk of finding occult invasive disease is reported to be as high as 28%. Routine use of core needle biopsy has been found to be accurate in diagnosing DCIS. However, sampling error may occur resulting in missed invasive disease in 10–20% of women [39–41]. Based on these data, the decision for axillary evaluation, specifically sentinel lymph node biopsy (SLNB), must be determined on an individual basis depending on the suspected risk of finding invasive disease at the time of final pathologic assessment of the surgical specimen. A recent review of axillary surgery practices in patients with DCIS evaluated 2159 women of whom 470 (22%) with high risk features completed a SLNB [42]. Of these, 43 were found to have a positive SLNB. When the sizes of the SLN metastases were evaluated, only 7 were larger than 0.2 mm. The remaining positive lymph nodes had only isolated tumor cells within the sentinel node. The authors conclude that a need for SLNB in the setting of DCIS exists but only in cases where high risk features are present and the risk of sampling error is significant. In practice, SLNB for DCIS is generally performed only when DCIS presents as a palpable mass or when mastectomy is being performed. 

## 7. Selection of Treatment

While no prospective randomized trial exists comparing mastectomy, breast conservation with radiation, and breast conservation without radiation for the treatment of DCIS, retrospective data suggest survival is similar among all methods and ranges between 98–100% [43, 44]. From this, it is accepted that treating DCIS is therefore not about survival but instead about limiting the rate of local recurrence. A multidisciplinary approach to DCIS is necessary for optimal patient evaluation and allows all data to be integrated in order to make clear treatment recommendations. Patient preference should also be factored into the treatment decision. 

Historical data demonstrates the risk of local recurrence after mastectomy is extremely low (<1%) (Table 1). Therefore, it remains an option for women who are not interested in or who have contraindications to breast conservation therapy. It is accepted as a standard therapy for DCIS but has been criticized based on the irony that an in situ disease that does not influence survival may be treated in some cases with a more radical surgical approach. Nonetheless, absolute and relative contraindications to breast conservation exist and include women in whom complete surgical clearance of tumor would result in unacceptable cosmesis, diffuse microcalcifications throughout the breast, the presence of a contraindication to radiation therapy, and patient preference.

## 8. Breast Conservation Therapy

When breast conservation is appropriate, the goals of the surgical procedure are total removal of the suspicious or known malignancy with minimal cosmetic deformity. Oncoplastic surgery, combining principles of oncologic surgery with plastic surgery techniques, has helped advance surgical excision of larger volumes of tissue, maintaining, oncologic principles while maintaining and sometimes improving cosmetic outcome [50]. 

The Early Breast Cancer Trialists Collaborative Group (EBCTCG) recently published a meta-analysis and overview of the DCIS prospective randomized trials treating women with breast-conserving surgery with or without radiation therapy [51]. The data continue to demonstrate no survival benefit from radiation therapy. However, it also continues to demonstrate the significant impact of radiation therapy in reducing local recurrence after breast-conserving surgery by 50–60%. Although lumpectomy alone is an accepted treatment for DCIS, rates of local recurrence are approximately 3% per year while the addition of radiation reduces this risk to approximately 1-2% per year. Interestingly, whether radiation therapy is given or not, 50% or more of all local recurrences after BCS for DCIS are invasive recurrences with recurrent DCIS making up the remainder. In depth analysis of the local recurrences in NSABP B-17 and B-24 by Wapnir et al. finds that mortality rates are significantly higher in women with a local recurrence of invasive breast cancer after BCS for DCIS [52].

**Figure 9:**
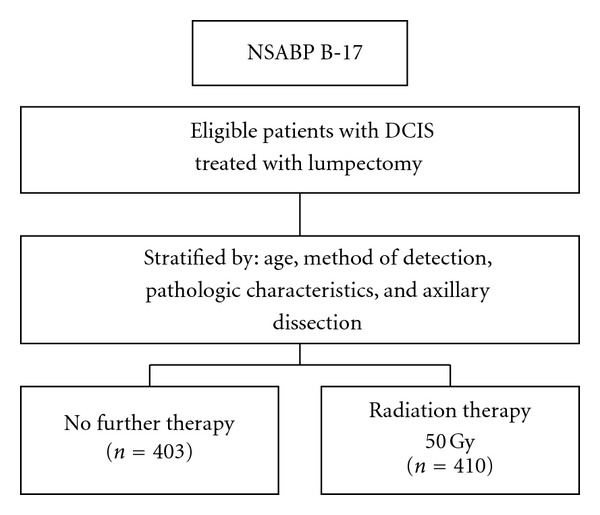
Diagram of NSABP-B17 trial.

The National Surgical Adjuvant Breast and Bowel Project (NSABP) B-17 trial is the first randomized, controlled trial for DCIS which confirmed the effectiveness of RT in decreasing local recurrence after lumpectomy with negative resection margins in mammographically or clinically detected DCIS [52–55] (Figure 9). 

The updated analysis with 15-year followup showed a decreased cumulative incidence of both invasive and noninvasive ipsilateral breast recurrences from 35% to 19.8% with the addition of radiation therapy. The incidence of invasive ipsilateral breast recurrence was also decreased from 19.4 to 8.9% with the addition of radiation therapy to lumpectomy [52] (Table 2). In the NSABP B-17, the annual mortality rate due to breast cancer in patients who had breast-conserving therapy was 0.67%. 

As confirmed by randomized trials, breast conservation including radiation therapy remains a standard treatment option for women diagnosed with DCIS (Table 3). Despite the results of randomized trials indicating the benefit of radiation therapy after conservative surgery, questions remain regarding the identification of a subgroup of patients who may not require radiation therapy after wide local excision.

The idea that some subgroups of women with DCIS may be appropriately treated without radiation therapy has been considered for well over a decade. Retrospective data, especially those of Silverstein et al. [35], have supported this hypothesis advocating that patients with a surgical margin of greater than 10 mm may be spared radiation with no change in their risk of local recurrence. More contemporary data published by Rudloff et al. [62] appears to support this as well, suggesting that there is no additional benefit to radiation therapy in patients with a margin >10 mm. Other published experiences have demonstrated variable recurrence rates with local excision alone (Table 4). 

To date, prospective data have failed to validate the elimination of radiation therapy from the treatment plan of women completing BCS for DCIS. Two prospective trials have attempted to identify a subset of patients with low-risk DCIS who may not benefit from the addition of radiation therapy following a local excision. One trial prospectively enrolled 158 patients with low-to-intermediate grade (LIG) DCIS with lesions ≤2.5 cm to treatment with a wide local excision with margins ≥1 cm followed by observation. This trial was closed to accrual after stopping criteria were met. With a median follow-up time of 40 months, 13 patients (8%) had developed a local recurrence [65]. The Eastern Cooperative Oncology Group (ECOG 5194) and the North Central Cancer Treatment Group (NCCTG) conducted a single-arm prospective study with 670 patients with either LIG DCIS measuring ≤2.5 cm or high-grade (HG) DCIS, measuring ≤1 cm who had microscopic margin widths of ≥3 mm and no residual calcifications on postoperative mammograms to determine the risk of ipsilateral breast events in patients with DCIS with local excision alone. A total of 670 patients enrolled were eligible for analysis. Patients enrolled in the year 2000 and later had the option to take tamoxifen. The 5-year rate of ipsilateral breast events in the LIG group was 6.1%, while the 5-year incidence for the HG group was 15.3% [66].

In comparison, Motwani et al. performed a retrospective review on 263 patients who met the eligibility criteria of the ECOG 5194 study and underwent breast-conserving surgery with or without adjuvant radiation. Five-year and 7-year ipsilateral breast tumor recurrence (IBTR) for the LIG cohort was 1.5% and 4.4% for patients treated with adjuvant radiation compared with the 6.1% and 10.5% with breast-conserving surgery alone, respectively. The 5-year and 7-year IBTR for the HG cohort was 2.0% and 2.0% with adjuvant radiation compared with 15.3% and 18% with breast-conserving surgery alone, respectively [67]. 

**Figure 10:**
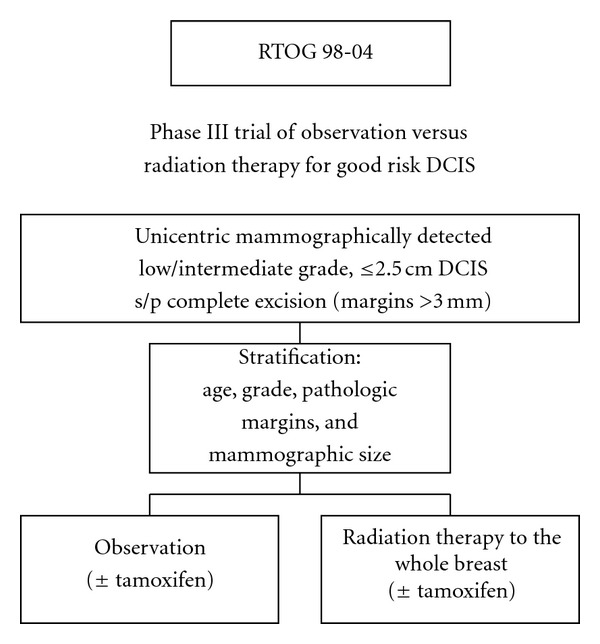
Diagram of RTOG 98-04 trial.

To further clarify the role of radiation therapy following excision in patients with low risk DCIS, RTOG recently completed RTOG 98-04, a trial randomizing low-risk DCIS patients to breast-conserving surgery with or without adjuvant radiation therapy (Figure 10). Initial results for RTOG 98-04 were reported in 2011, which included a subset of women with mammographically detected LIG DCIS, size ≤2.5 cm, and margins ≥3 mm. Tamoxifen use for 5 years was allowed but not required. Monthly accrual numbers were not met, therefore the study was closed early. A total of 593 women were randomized to breast-conserving surgery followed by whole breast radiation totaling 5000 cGy or to observation. The median followup was 6.46 years. Local failure at 5 years was 0.4% for women treated with radiation versus 3.2% of women who were observed (*P* = 0.0023). Size, grade, margin status, and age had no impact on local failure. In the observation arm, local failure with tamoxifen was 3% versus 8.9% without tamoxifen. Contralateral breast failures were similar in both arms at 3.6% with tamoxifen use and 2.7% without. Both regimens were well tolerated and the disease-free survival and overall survival were not different between the two arms. The local failure rate of 3% compared favorably with the 6.1% local failure at 5 years in the ECOG 5194 observation trial, which may reflect the increased use of tamoxifen. In the future, clinical trials for this subset of women may include endpoints, such as, acute and chronic sequelae of local and systemic therapy, assessment of cosmetic results, and the economic impact of the cost of therapy. Inclusion of these data may allow us to better inform this subset of patients about the risks and benefits of adjuvant therapies. 

## 9. Hypofractionation

Hypofractionation has been investigated extensively for invasive carcinoma but whether hypofractionation is as effective for pure DCIS has yet to be established. In a retrospective review, Wai et al. found hypofractionation to be as effective as standard fractionation [68]. The Trans Tasman Radiation Oncology Group (TROG) has recently initiated a prospective international clinical trial randomizing patients with pure DCIS to hypofractionated versus standard fractionated whole breast RT, with or without a partial breast boost. 

## 10. Partial Breast Irradiation

The majority of local breast tumor recurrences occurs in proximity to the original primary tumor site [69, 70]. In addition, the incidence of local recurrences elsewhere in the breast is equivalent in women treated with or without radiation therapy after a lumpectomy [71]. As a result, there is increasing interest in partial breast irradiation techniques to treat the lumpectomy cavity alone with radiation as opposed to whole breast radiation. Early experience with partial breast irradiation in patients with DCIS suggests outcomes similar to conventional whole breast radiation therapy (Table 5).

 Partial breast irradiation can be delivered through multiple techniques, including multicatheter brachytherapy, intracavity brachytherapy, partial breast external beam radiation therapy, or intraoperative radiation. The typical treatment course for brachytherapy and external beam therapy consists of 3400 cGy in 10 fractions or 3850 cGy in 10 fractions delivered twice a day. Intraoperative radiation is typically delivered in 1 dose of 1000–2000 cGy. 

**Figure 11:**
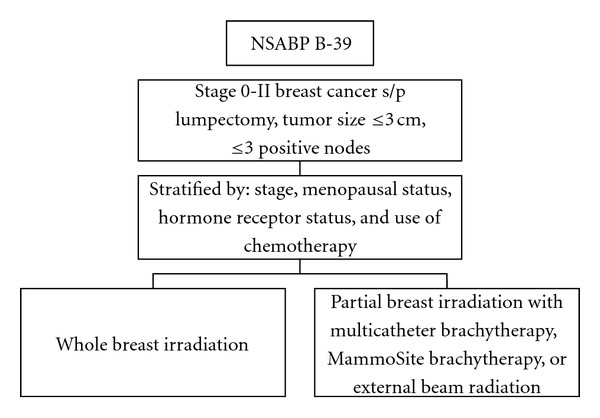
Diagram of NSABP B-39 trial.

The NSABP is currently conducting a randomized, Phase III trial, B-39, to test the equivalency of partial breast irradiation by randomizing patients with Stage 0-II breast cancer status after a lumpectomy to whole breast radiation or partial breast radiation (Figure 11). Until NSABP B-39 has completed accrual and follow-up results are available, physicians are encouraged to use caution in selecting appropriate patients for partial breast irradiation and discuss the potential uncertainties with this technique. The American Society for Therapeutic Radiology and Oncology (ASTRO) has published consensus guidelines for treatment of patients outside of clinical trials [76].

## 11. Oncotype DX

Recently, Solin performed a prospective validation study using the Oncotype DX assay on DCIS tumor specimens from 327 patients from ECOG 5194. Their results were presented at the San Antonio Breast Cancer Symposium, validating that this multigene assay quantifies recurrence risk and complements traditional clinical and pathologic factors in selected patients with DCIS treated with surgical excision without adjuvant radiation [77]. This provides us with a new tool to help predict women at higher risk for local recurrence, but further evaluation is necessary to see how it will be incorporated into practice.

## 12. Endocrine Therapy

**Figure 12:**
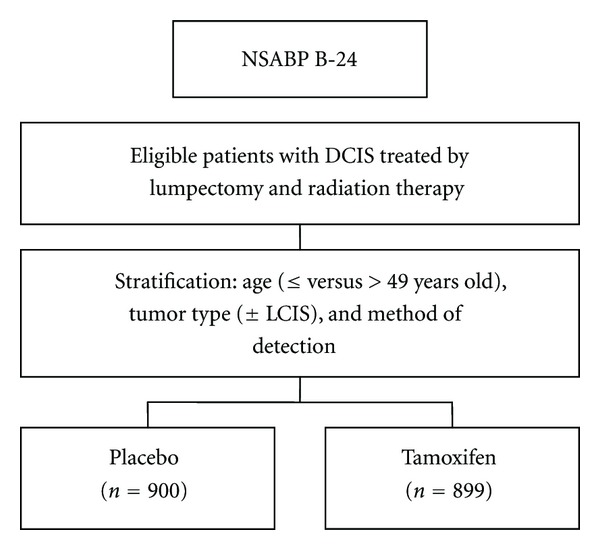
Diagram of NSABP B-24 trial.

The NSABP has conducted a double-blind randomized, controlled trial, NSABP B-24, to examine the potential benefit of tamoxifen in patients with DCIS who were treated with lumpectomy and radiation therapy [78] (Figure 12). Women were randomized to either tamoxifen or placebo for 5 years following breast-conserving therapy. With 15-year followup, tamoxifen reduced the risk of ipsilateral invasive breast tumor recurrence by 32% in patients treated with excision plus radiation therapy [55]. Noninvasive ipsilateral breast recurrence was a nonsignificant 16% reduction in risk. Tamoxifen also reduced the risk of contralateral breast cancer development by 32%. No differences in distant disease, breast-cancer-specific survival, or overall survival were found between those patients treated with or without tamoxifen. Tamoxifen can cause life-altering side effects, such as, thromboembolic disease and uterine cancer and is not without potential serious toxicity. The role of tamoxifen in the treatment of patients with DCIS remains uncertain.

As a result of these potential toxicities, as well as the beneficial effect of aromatase inhibitors in the adjuvant treatment of hormone-responsive invasive breast cancer, the NSABP is conducting the B-35 trial designed to compare the effects of tamoxifen and an aromatase inhibitor, anastrozole, on the occurrence of local, regional, distant, or contralateral breast cancer. Postmenopausal women with DCIS, estrogen receptor or progesterone receptor positive, who completed local excision, were randomized to radiation therapy plus 5 years of ongoing treatment with either tamoxifen or anastrozole. The study has completed accrual and may provide additional choices in the treatment of women with DCIS when long-term follow-up results are available.

## 13. Radiation Therapy

**Figure 13:**
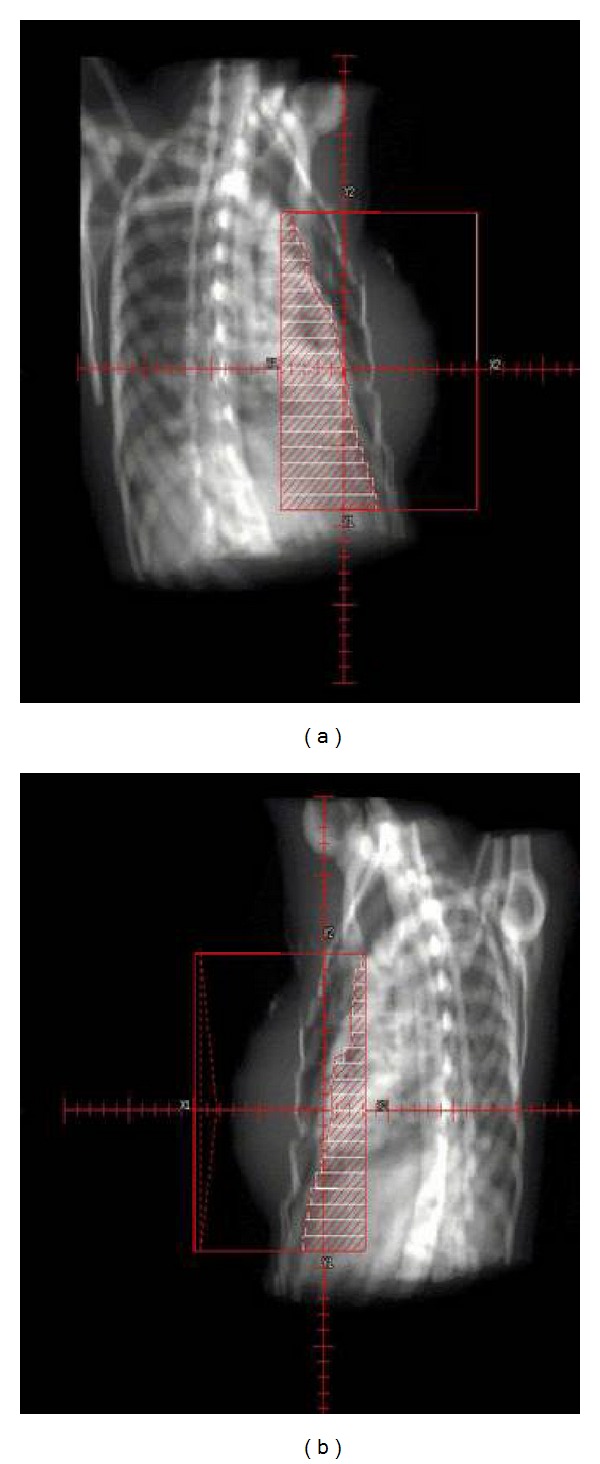
Standard tangential breast radiation therapy, medial and lateral fields.

Radiation therapy should be delivered after a complete assessment of surgical and pathologic findings, as well as a postoperative mammogram to verify no residual suspicious abnormalities remain. Radiation therapy for DCIS generally consists of treatment to the ipsilateral breast without inclusion of the regional lymph nodes (Figure 13). 

**Figure 14:**
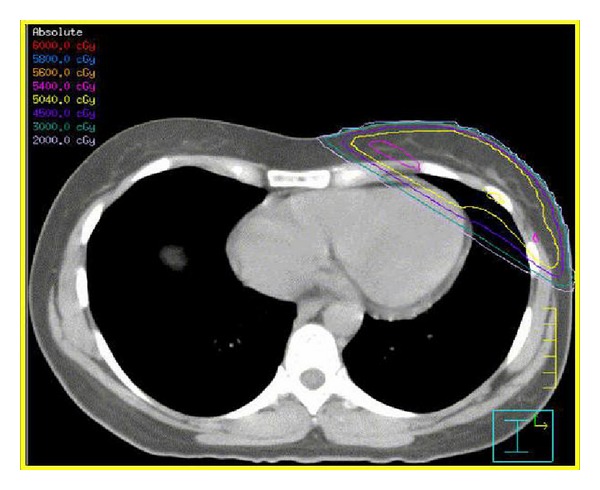
Radiation therapy planning CT with isodose distributions.

The radiation oncologist simulates the treatment field and modern planning is performed with CT-based treatment planning to determine adequate dose homogeneity within the target (Figure 14). CT-based treatment planning also ensures there is minimal dose to the ipsilateral lung and heart.

Standard whole breast radiation is performed through opposed tangential fields on a daily basis, Monday through Friday. Dose is 180–200 cGy per day for a total dose of 4500–5000 cGy, typically delivered over 5–5.5 weeks.

Controversy exists regarding the role of boost irradiation in DCIS. Tumor size, grade, and margin status are often taken into consideration when considering additional dose delivered through a tumor bed boost. In a study of 220 patients with DCIS, 79 patients received a boost, the majority of whom frequently were classified into higher risk categories as defined by the Van Nuys Prognostic index. Of these 79 patients, none of the patients who received a boost developed a local recurrence compared with 5.7% of patients who did not receive a boost, suggesting a role for radiotherapy boost to the surgical cavity [79]. 

## 14. Followup

Patients should have close followup including physical examination every 6 months for at least 5 years to detect recurrent or new primary tumors. Evaluation should include an overall cosmetic result assessment and identification of any acute or chronic sequelae of treatment. Routine tests, such as, bone scan, chest X-ray, CT scan, and liver function tests are not indicated for asymptomatic patients treated for DCIS.

Postsurgical and postradiation changes including skin thickening, edema, and fluid collections will be most marked in the first 6 months. For most patients, radiographic changes will slowly resolve within 2 years of treatment. Mammogram of the treated breast should be performed every 6 months or at more frequent intervals as warranted by clinical or radiographic findings. This schedule should continue until postoperative and postradiotherapy changes have stabilized as judged by a radiologist specializing in breast imaging. Annual mammography of the contralateral breast should continue to be performed according to the guidelines endorsed by both the American College of Radiology and the American Cancer Society. 

## 15. Recurrence

Of the recurrences that occur after primary treatment for DCIS, approximately one-half to two-thirds are cases of invasive cancer [52]. Although no consensus exists, most authors recommend mastectomy for patients with recurrence if breast-conserving therapy was the initial treatment. Systemic therapy is recommended based on standard prognostic factors, such as, nodal and hormonal status to predict the risk for metastasis. 

## 16. Conclusions

DCIS represents a heterogeneous pathologic condition. The incidence of DCIS continues to increase and is most frequently discovered on imaging. Consistent pathologic evaluation is crucial in optimizing treatment recommendations. Surgical treatment options include breast-conserving surgery and mastectomy. Breast-conserving surgery followed by postoperative radiation therapy is considered the standard of care with large randomized trials demonstrating a decrease in the incidence of local recurrence with the addition of radiation therapy. Further study is necessary to determine if a subset of patients with DCIS may require only surgery alone without adjuvant therapy. The optimization of diagnostic imaging, treatment with regards to pathological risk assessment, various irradiation techniques, and the role of endocrine therapy continue to evolve.

## Figures and Tables

**Table 1:** Results of treatment of DCIS with mastectomy.

Study	Number of patients	Followup (months)	Number of recurrences
Sunshine et al. [45]	68	120	0
Farrow [46]	181	60	2
Silverstein et al. [47]	228	84	2
Kinne et al. [48]	101	138	1
Schuh et al. [49]	51	66	1
Arnesson et al. [5]	28	77	0

**Table 2:** NSABP B17: ipsilateral breast tumor recurrence.

NSABP B-17	Number of events	Rate/1000 patient/year	Relative risk	*P* value
Noninvasive				

Lumpectomy alone	62	14.7	0.47	<0.001
Lumpectomy + RT	37	7.5		

Invasive				

Lumpectomy alone	79	18.8	0.52	<0.001
Lumpectomy + RT	44	9.0		

**Table 3:** Results of treatment after breast-conserving therapy for DCIS.

Study	Number of patients	Followup (months)	Number of recurrences (%)
Kurtz et al. [56]	44	61	3 (7)
Kuske et al. [57]	70	48	3 (4)
Silverstein et al. [58]	103	45	10 (10)
Solin et al. [59]	268	124	45 (17)
B17, Wapnir et al. [52]	410	207	81 (20)
B24, Wapnir et al. [52]	900	163	149 (17)
Bijker et al. [60]	507	126	75 (15)
UKCCR [61]	267	53	15 (6)

**Table 4:** Results of DCIS treated with excision alone.

Study	Number of patients	Followup (months)	Number of recurrences (%)
Lagios et al. [63]	79	44	8 (10)
Silverstein et al. [58]	26	18	2 (8)
Schwartz et al. [64]	72	47	11 (15)
B17, Wapnir et al. [52]	403	207	141 (35)
Bijker et al. [60]	503	126	132 (26)
UKCCR [61]	544	53	97 (18)

**Table 5:** Initial recurrence rates after partial breast irradiation for DCIS.

Study	Number of patients	Followup (months)	Number of recurrences
Benitez et al. [72]	100	9.5	2 (2%)
Chao et al. [73]	23	22.1	1 (4%)
Jeruss et al. [74]	194	54.4	7 (3.6%)
Benitez et al. [75]	36	66.0	0 (0%)
